# Supplementary material for: Interval Cancer Rate and Diagnostic Performance of Fecal Immunochemical Test According to Family History of Colorectal Cancer
Source: J Clin Med. 2020 Oct 14;9(10):3302. doi: 10.3390/jcm9103302 (PMC7602405; doi:10.3390/jcm9103302)
Supplement: Supplementary file 1 [file jcm-09-03302-s001.pdf]

Supplementary information

Table S1. The proportion of qualitative and quantitative FITs

	Family History of CRC N = 224,178	No Family History of CRC N = 5,419,260
Qualitative FITs	159,398 (71.1%)	3,716,171 (68.6%)
Quantitative FITs	64,780 (28.9%)	1,703,088 (31.4%)
Missing data	0 (0.0%)	1 (0.0%)

FIT, fecal immunochemical test

Table S2. Participation rate of colorectal cancer screening (FIT) in Korea, 2009-2011

	2009	2010	2011
No. of invitations	8,483,437	9,075,852	9,271,231
No. of participants	2,281,444	2,794,663	3,049,112
Screening rate	26.9%	30.8%	32.9%

FIT, fecal immunochemical test
The above numbers are based on a previous paper (reference No. 23).
